# Highlights from the 2013 Science of Placebo thematic workshop

**DOI:** 10.3332/ecancer.2013.346

**Published:** 2013-09-04

**Authors:** Marco Annoni

**Affiliations:** University of Milan, Department of Health Sciences, Milan 20142, Italy; European School of Molecular Medicine, Milan 20139, Italy; European Institute of Oncology, Milan 20141, Italy; FOLSATEC, Milan 20139, Italy

**Keywords:** placebo, placebo effect, placebo response, pain, chronic pain, depression, Parkinson, fMRI, PET, randomisation, clinical trial, acupuncture

## Abstract

In the last 30 years, a converging series of laboratory experiments, clinical trials, and neurocognitive studies have identified several key mechanisms of placebo effects. These studies suggest not only that placebo responses may be ubiquitous across research and clinical settings, but also that they can significantly modulate symptoms across a wide spectrum of highly prevalent conditions such as acute pain, chronic pain, anxiety, depression, Parkinson’s disease, and nausea, just to name a few. In order to inform the medical community about the most recent advances in the field of placebo studies, a thematic workshop entitled “The Science of Placebo” was held at the Beth Israel Deaconesses Medical Center (BIDMC), Harvard Medical School, in Boston (MA), on the 19–20 of June 2013. The workshop, sponsored by The Robert Wood Johnson Foundation, was organised by the Program in Placebo Studies and the Therapeutic Encounter, a Harvard-wide network of researchers dedicated to the study of the placebo phenomenon hosted by the BIDMC. The event was structured as a series of four public lectures, each delivered by a leading investigator in the field of placebo studies. The four keynote speakers were Fabrizio Benedetti, professor of neurophysiology and human physiology at the University of Turin Medical School and at the National Institute of Neuroscience in Italy; Tor Wager, director of the Cognitive and Affective Control Laboratory and associate professor of psychology and neuroscience at the University of Colorado; Predrag Petrovic, psychiatrist and researcher in the Department of Clinical Neuroscience at the Karolinska Institute in Stockholm; and Ted Kaptchuk, director of the Program in Placebo Studies and associate professor of medicine at Harvard Medical School.

## Introduction

Placebos occupy a special niche within contemporary medicine. Historically, healers and physicians have always been aware of the ‘placebo effect’, the fact that psychological and contextual factors may concur in shaping how people react to therapy alongside real and effective interventions. In the *Charmides*, Plato noted—through Socrates’ voice—that to soothe a headache, one needed ‘a kind of leaf, which required to be accompanied by a charm, and if a person would repeat the charm at the same time he used the cure, he would be made whole; but that without the charm would be of no avail’. For more than two millennia, this ‘charm’ remained one of the few weapons at the healers’ disposal. Before the emergence of science-based medicine, there was no objective way of assessing the effects of medical interventions. Still, in 1930 only a dozen of compounds were effective, while many were useless, some were dangerous, and a few lethal. To a large extent, then, the history of pre-scientific medicine has been the history of placebos or the history of ‘fake medicines’.

After World War II, two factors conspired in changing how physicians, researchers, and society understood placebos and their effects. The first was the systematic introduction of randomised controlled trials as the gold standard of clinical research. Soon the word ‘placebo’ was adopted to name the sham interventions used as controls in clinical trials. Accordingly, the ‘placebo effect’ began to be understood as a confounding factor among many others, a subjective background noise requiring blinding and appropriate controls rather than explanation. The second factor was the rise of autonomy-based accounts within medical ethics. Starting in the early 1960s, clinical ethicists began to target the practice of administering placebos for ‘the good of the patient’ as a clear case of a no longer justifiable medical paternalism. By the end of the 1970s, the word ‘placebo’ was synonymous with a ‘fake intervention’ deceptively administered in clinical trials as a control or in clinical settings to unethically mollify patients.

This situation remained virtually unchanged until a converging series of laboratory experiments, clinical trials, and neurocognitive studies began to uncover the underlying mechanisms of placebo effects. Slowly, the placebo phenomenon ceased to be a curiosity at the fringes of medicine and started to become a worthy target of empirical research. Today one can find more than 2,000 research papers on PubMed explicitly dedicated to placebo ‘effects’ or ‘responses’; although the first studies date to more than 30 years ago, the number of studies has exploded in the last decade. Collectively, what these empirical studies suggest is not only that placebo responses may be ubiquitous across research and clinical settings but also that they can significantly modulate symptoms across a wide spectrum of highly prevalent conditions such as acute pain, chronic pain, anxiety, mild depression, and nausea, to name a few. These significant discoveries, however, have not corresponded to an equally significant advance in how placebo effects have been conceptualised. Today substantial disagreement persists even as to how we should define ‘placebo effects’. While many placebo mechanisms have been identified, it is still unclear whether they can all be reduced to a unique model and thus understood as diverse instances of a general phenomenon. Clearing the field from these fundamental questions, however, is a necessary pre-condition to generate further hypotheses to be tested, to evaluate the possible clinical applications of this knowledge, and finally to assess what the implications at the regulatory level can be. If placebo studies are to live up to the expectation of being a game-changer for healthcare, they must be able to reclaim their place as a self-aware and coherent area of scientific inquiry, medical practice, and policy-making.

With this objective in sight, the workshop held on 19–20 of June 2013 at Harvard Medical School in Boston (MA), and entitled ‘The Science of Placebo’, was like taking a first but significant step. The workshop was organised by the Program in Placebo Studies and the Therapeutic Encounter (PiPS), a research center dedicated to the study of the placebo phenomenon that draws on faculty and researchers across Harvard Medical School and is hosted by the BIDMC. The PiPS’s mission is to examine ‘the biological basis of the placebo response [in order] to elucidate, quantify, and optimise the “non-specific” dimensions of health care’, thus improving the effectiveness of clinical care and the efficiency of pharmaceutical development. The Robert Wood Johnson Foundation (RWJF) sponsored this workshop as a part of a grant aimed at informing the medical community about the most important advances recently made in the field of placebo studies. The goal of RWJF was to broaden discussion on placebo to include a wider audience and move awareness of placebo research from the margins of biomedicine to a central concern. It is also hoped that a wider dissemination will influence medical education. Harvard Medical School sponsored it as an official course for academic credit. This event is the first in a series of five. The next symposium will take place on 9 December 2013 at Massachusetts General Hospital, under the title ‘Where is the placebo in shared decision-making and clinical guidelines?’; the third event is scheduled for 11 February 2014, and it will focus on ‘Healing and placebos: medicine, religion and rituals’. The titles and locations of the last two symposia are yet to be decided.

This first workshop has been structured as a series of four public lectures, each delivered by a leading investigator in the field of placebo studies. The four keynote speakers were Fabrizio Benedetti, professor of neurophysiology and human physiology at the University of Turin Medical School and at the National Institute of Neuroscience in Italy; Tor Wager, director of the Cognitive and Affective Control Laboratory and associate professor of psychology and neuroscience at the University of Colorado in the United States; Predrag Petrovic, psychiatrist and researcher in the Department of Clinical Neuroscience at the Karolinska Institute in Stockholm in Sweden; and Ted Kaptchuk, director of the Program in Placebo Studies and Therapeutic Encounter and professor of medicine at Harvard Medical School. Over 400 physicians, researchers, educators, and students attended the event. What follows is a report of the four lectures that were presented over this two-day event.

## Fabrizio Benedetti: ‘Placebos, words, and drugs: what’s the difference?’

In the first public lecture, Fabrizio Benedetti focused on the difference between placebo and drug effect and on its significance for neurology. When a drug is administered to a patient, noted Benedetti, a clinical improvement may take place. This improvement may be due to the specific effect of the drug or due to other factors, such as the spontaneous remission of the disease, statistical artefacts such as regression to the mean, subjective reporting bias, and to the psychosocial context surrounding the therapeutic act. While the specific effects of a drug are usually explained in terms of specific molecules binding to receptors in the brain, traditional clinical trials are not suited to studying the effects of social and sensory stimuli on neurocognitive systems. To isolate and measure the various components of the therapeutic ritual, researchers need to replace the active drug with a placebo in controlled experiments. By eliminating the specific pharmacological component of the drug, neurologists may study how such variables operate at the level of fundamental neurocognitive mechanisms. Hence, placebo responses may provide a useful model for understanding how the brain works in many conditions.

Over the last three decades, two general conclusions have emerged out of this experimental approach. First, there is not a single ‘placebo effect’; rather there are several placebo responses that may recruit different mechanisms and systems across the body, including the opioid and cannabinoid systems, the dopaminergic pathways, and the endocrine system. Second, placebo responses operate by activating the very same biochemical pathways that are activated by drugs in routine medical practice. To support and further explore the many implications of these hypotheses, in the central part of his talk, Benedetti discussed a few studies that he has conducted with his collaborators on placebo responses in pain and Parkinson’s disease.

Pain is the area that has been most investigated in placebo studies. In a study [1], Benedetti and colleagues demonstrated how complex the interaction is between prior experiences, expectations, and pain modulation, so that a single mental event (e.g., conscious expectations) may lead to the same effect (pain analgesia) by recruiting diverse systems (endogenous opioid or endogenous cannabinoid system) depending on prior drug exposure. The experiment involved ischaemic arm pain induced with an inflatable sphygmomanometer cuff. On day 1, researchers measured baseline pain tolerance. On days 2 and 3, participants received either an opioid drug (morphine) or a cannabinoid drug (ketorolac), and they were then exposed to the same pain stimulus. Predictably, both drugs led to a significant increase in pain tolerance. On day 4, participants underwent the same procedure, but they received a placebo instead of the real drug. Interestingly, a significant analgesic effect was observed in both groups, and this effect correlated with the kind of analgesic drug previously administered. By coupling naloxone (a μ opioid receptor antagonist) with the placebo in the morphine group, placebo analgesia was selectively inhibited, demonstrating that such an effect relied on the activation of endogenous μ opioid receptors. Likewise, coupling the placebo with a CB1 receptor antagonist, such as rimonabant, could selectively inhibit the analgesic effect observed in the ketorolac group. Coupling the placebo with naloxone in the ketorolac group – or the placebo with rimonabant in the morphine group – did not inhibit placebo analgesia, demonstrating that such effects depended on two diverse endogenous mechanisms. The resulting model is that expectation about therapeutic benefit, a complex mental phenomenon, can produce a real analgesic effect via different neurochemical systems. Expectation is thus a sort of ‘switch’ for the selective activation of either endogenous opioid or cannabinoid receptors depending on prior drug exposure.

Parkinson’s disease is another useful model in understanding the neurobiology of placebo responses. Here Parkinson’s disease is understood as a movement disorder, and placebo effects are measured in terms of improvement of motor performances. In a second study, Benedetti and colleagues compared an anti-Parkinson drug (apomorphine) with a placebo [2]. Interestingly, they observed a similar response at the clinical and the electrophysiological level, with placebo effects leading to a similar decrease in muscle rigidity and in single neuron firing rate in the subthalamic nucleus (recorded through electrodes implanted during deep brain stimulation). While this study suggests that placebos and anti-Parkinson drugs may have similar effects and rely on similar mechanisms, it does not follow that such effects are identical. In fact, in Parkinson’s motor disorder placebo and drug responses differ as to duration of the effect, individual variability, and overall magnitude. In comparison with apomorphine, placebo effects do not last anywhere near as long, have a higher rate of non-responders, and lower magnitude [3]. Hence, although the underlying mechanisms are probably the same (decrease in neurons firing rate and release of dopamine in the striatum), significant differences exist between placebo and drug administration. In addition, studies on placebo responses in Parkinson’s disease also support the conclusion that two key factors may play a major role in eliciting placebo responses. The first is genetic predisposition, for example polymorphisms that affect dopaminergic pathways. The second variable is learning. While many do not respond to the first placebo exposure, after repeated administration of successful treatments, virtually anyone becomes a responder.

Benedetti ended his lecture by illustrating one of the many clinical implications deriving from this model of placebo responses. According to his view, different social and sensory stimuli in a therapeutic encounter can activate the release of molecules in the brain (such as endogenous opioids, endogenous cannabinoids, or endogenous dopamine) that bind to the very same receptors of real drugs. This raises the question of how drug-specific effects may interact with the effects triggered by therapeutic rituals in clinical settings. Is there a possible interference? What happens if one strips away all the rituals from the administration of morphine or other drugs? What are the effects for clinical practice? A preliminary answer to these questions comes from open-hidden experiments in which the same treatment (e.g., an analgesic such as metamizol) is delivered either covertly (e.g., by automatic intravenous infusions delivered by a random machine) or in the full view of a real physician as in routing clinical practice [4]. By using this trial design, it was possible to demonstrate that a placebo administered in full view of the patient had an analgesic effect roughly equivalent to the one of an effective painkiller such as metamizol administered covertly. This conclusion, now supported by other studies, suggests that placebo responses may play a significant role in clinical practice and in other contexts, such as physical performances and extreme conditions, where the same underlying mechanisms may be at stake.

## Tor D. Wager: ‘Studying the person: brain mechanisms of placebo analgesia’

Wager began his lecture by asking whether there is evidence that entertaining a certain belief can be helpful to relieve pain. Besides the results presented by Benedetti, today many studies support an affirmative answer to this question. Recently, a striking example has been provided by the so-called German acupuncture trials (GERAC) [5]. This double-blind, placebo-controlled, multi-centred, parallel-group trial with 1,162 patients aimed at comparing traditional therapy (drugs and exercises) for chronic low-back pain against *verum *acupuncture and sham acupuncture (performed with retractable needles). Interestingly, this large evidence-based study found that acupuncture was almost twice as effective as the standard conventional therapy. Even more importantly, researchers found that between *verum *and sham acupunctures, there was virtually no difference. Hence, in this case, a sham procedure proved to be more effective than the standard therapy. These results may suggest that something is wrong with the current standard of care, but a more plausible and interesting conclusion is that healing rituals may have a real and significant effect on highly prevalent conditions such as chronic low-back pain.

According to Wager, neuroscience can contribute in two ways to further elucidate the power of therapeutic rituals. One is to provide evidence for the plausible mechanisms of placebo effects. Which brain systems are involved? Where and how should we intervene in the system to understand the experience of pain, and how is the system to be further analysed and studied? Second, they can provide intermediate markers, that is ways of identifying and measuring phenomena in the brain that are useful to understand how placebo responses affect pain and other conditions, how profound these effects are, and what their limitations are. In turn, this would yield important clues to develop more individualised and effective treatments.

As for pain, a first step in this direction has been to enquire whether or not placebo treatments really affect the brain mechanisms of pain. In their first study with fMRI on placebo effects, Wager and colleagues compared two other studies, looking into whether there were consistent effects across them [6]. In one experiment, researchers manipulated participants’ expectations so as to convince them that they were being administered with the same intense painful stimulus (in fact it was just a mild one) on two different spots on the same arm: one covered with a control inert cream and the other with a powerful analgesic cream. In reality, the latter cream was a placebo. In this way, each person served as her own control. The goal of the trial was, first, to test whether the manipulation of expectations caused by different verbal instructions could induce a localised placebo analgesic response and second, whether such a response could be consistently associated with some neural correlates. The difference in reported pain and brain activity was the measure of the placebo effect. This study found a significant placebo analgesic effect in reported pain and robust brain activation. In particular, a reduction in brain responses to heat was observed in key parts of the pain processing system, including the rostral anterior cingulate cortex (rACC), the insula, and the thalamus. Together, these results suggested a positive correlation between the subjective reports and the decrease of brain activity in these regions, so that the larger the drop in reported pain is, the larger the analgesic effect, and the larger the drop in brain activity.

Over the years, other studies have independently supported the conclusions of this research, increasing the experimental data available on the correlations between the activation of key brain areas, such as the lateral prefrontal cortex—which is involved during the anticipation of pain—and the midbrain periaqueductal gray (PAG)—which is known to be involved in the opioid system. Building on these results and on other studies, Wager then explained how recent meta-analyses looking for consistent correlations have identified the ACC, the medial thalamus, and the insula, as other areas of interest [7]. All of these areas have been individually and collectively linked to the genesis of the feeling and suffering associated with pain As a first interim conclusion, Wager stated that placebo treatments do have consistent effects on likely pain mechanisms across different studies.

A second key question relates to the mechanisms that link expectation to placebo effects. As Benedetti showed, placebo treatments may induce many placebo effects that can translate into changes in emotions, associative learning (or conditioning), evaluation, and so on [8]. Although all of these mechanisms have to be unpacked to gain a better understanding of placebo responses, Wager and his research group focused their attention especially on associative learning and evaluation processes. There are many potential mechanisms of conditioned placebo responses, but there are two candidates for the role of central mediators of a variety of expectancy effects: a frontal-parietal system that is important to maintain cognitive contexts (i.e. knowing where you are and what treatment you are getting now); and a distribute system involving the ventromedial prefrontal cortex (VMPFC), the orbitofrontal cortex (OFC), the thalamus, the nucleus accumbens, and the PAG. This latter system is the one involved in evaluation tasks and, in particular, in learning new values. Studies by Wager and others reported results consistent with the hypothesis that both systems are important and may interact together in placebo responses [9, 10]. The theory emerging from these studies is that placebo effects may involve multiple brain systems and mechanisms, but some are particularly important, especially those linking situational context first to meaning and evaluation and then to particular effectors system.

This view indicates a possible strategy to answer a third crucial question, namely, whether or not there are common mechanisms for placebo responses across diverse disorders. If the theory that value learning is an essential component of placebo responses is correct, it is possible to generate a series of empirically testable hypotheses. For example, assuming this view, we should expect that placebo effects are largest in those disorders that involve affective meaning and evaluation circuitry, similar to Parkinson’s disease and depression. Parkinson’s disease is an interesting case because it is a neurological and not just a psychiatric disorder, and yet it depends critically on evaluation circuitry involved in learning processes. While this hypothesis is complementary to the Benedetti results, it also emphasises the role of reward systems to account for placebo responses in Parkinson’s disease. Preliminary studies on placebo and Parkinson’s disease complement this view by showing that an increase in dopamine is correlated with reward only. These results lead to the conclusion that value-learning mechanisms may provide a key to understanding how placebo effects are created and maintained in many conditions.

Reinforcing the final point previously made by Benedetti, Wager identified as one of the core challenges for the future of placebo studies the elucidation of the possible interactions between the effects generated by contexts—as they are partially perceived and partially constructed by the brain—and the specific effects of pharmacological intervention. The goal is to understand whether it is possible to exploit synergistically these various effects, thus enhancing therapy while reducing drug intake and side effects. This opens a new series of interesting questions that need to be tested with a number of psychological treatments and with different drugs. In the future, we need to test pharmacological and psychological treatments jointly, instead of only considering the drug or only the placebos as the sole relevant dimension of care delivery.

## Pedrag Petrovic: ‘Neurobiology of placebo and delusion’

Petrovic began his talk by putting forward the view that placebo effects are interpretable as the results of the brain processing information. This perspective provides the starting point to construct a unitary model to understand how expectations affect analgesia and may interact with delusion and psychosis. For several hundred years, it has been known that humans have a nervous system that transmits signals to the brain. Traditionally, the crucial question was whether the brain is just a mirror of the external world or whether the external world is something completely different in comparison with our experiences. Today cognitive neuroscience indicates that it may be something in between these two extreme poles. We process external signals, but we also have expectations, values, and goals that shape the way in which we make sense of the external world. Optical illusions—like the ‘hollow mask effect’—illustrate well how social stimuli may shape our perception. Expectations are thus very important to determine how we interpret the world. Importantly, expectations can also be experimentally manipulated.

These general remarks are important insofar as expectations are also known to have a crucial role in determining placebo responses in at least pain, mild-to-moderate depression, and Parkinson’s movement disorder. The driving hypothesis, thus, is that the same underlying mechanisms are responsible for different placebo effects across different conditions inasmuch as expectations are involved in all of them, even though relevant differences may also be present. In this talk, Petrovic showed how evidence coming from converging sources is backing up such a claim with empirical data.

As for pain, this is the scientific area in which scientists have learned the most about placebo effects. Among other things, empirical research on pain and placebo has led to two conclusions. First, manipulation of expectations of treatment is very important. Typically, a standard trial design involves three phases: (i) a pre-phase in which pain is experienced and assessed, (ii) a manipulation phase in which a sham treatment is administered while the pain is artificially decreased in order to induce positive expectations, and (iii) a subsequent phase in which the placebo alone leads to perceive the pain as less intense. Importantly, if expectation manipulation is not performed (e.g., by telling the subjects that ‘this is a powerful painkiller’), no reduction in pain perception is observed. The other thing shown by studies on placebo analgesia is that the endogenous opioid system is also very important. First, Levine [11] and then Amanzio and Benedetti [1] showed that by pre-treating a patient with naloxone, it is possible to effectively suppress placebo analgesia, thus indicating that such a response relies on the activation of endogenous opioids. Further studies with PET [12] showed that opioid analgesia leads to a large activation of two brain regions: the ACC and the lateral OFC. Although other areas may be involved in placebo responses—as shown also by Wager—Petrovic believes that these two areas are of primary importance.

If pain is an important area of research, Petrovic argued, understanding placebo effects in depression and anxiety may yield even more significant results. The reason is that a large meta-analysis indicates that the difference between antidepressants and placebos for moderate-to-mild depression can be very tiny [13]. Yet, in order to understand whether there is a real placebo effect at stake in emotion regulation, how strong it is, and what its driving factors are, what is required is to understand how we can modulate people’s expectations in emotionally loaded situations. Is it possible to change the way people perceive emotional pictures by manipulating their expectations? To answer this question, Petrovic and his colleagues [14] conducted a trial on the ‘emotional placebo’ in which unpleasant images were presented to people who had to rate how much unpleasantness they experienced. Subjects were then treated with a small dose of intravenous benzodiazepine, which led to a decrease in the rated unpleasantness. The next day they repeated the same procedure in a PET scanner. People were presented with similar pictures, but this time they were treated only with a saline solution (placebo). The study detected a decreased activity in the network processing emotional signals – including the amygdala. More interestingly, they observed an increased activity in the same regions identified by the previous study on the placebo analgesia: the rACC and the lateral OFC. Hence, at least at the level of functional activation, it is possible to generalise the idea that there are common mechanisms that operate in both analgesic and emotional placebo responses.

But if placebo responses activate the same brain regions across different conditions, the next question to address becomes what the functions of these brain regions are. As for the rACC, this area has been consistently linked to the opioid system and to placebo analgesia, with PET studies revealing a large concentration of opioid receptors in this area. Generally, the ACC is recognised as being important for attention-oriented tasks. Also, meta-analyses also suggest that a significant difference exists, as more cognitive tasks activate the codal part of this region, while more emotional ones activate its rostral part. As to the OFC, this region has been shown to be involved in complex emotional regulation processes, for example reappraisal. Volume differences have also been observed, with people more in control of their emotions having larger orbitofrontal regions. But this area is also important to construct rules, to regulate goal-driven behaviour finalised to homeostasis, and to process expected reward and aversive error signals, that is, those signals generated by a mismatch between what is expected and what actually occurs [14–16].

If both of these regions are important in placebo effects, they should work together. Petrovic and his colleagues tested this hypothesis in the two studies described above. In the case of placebo analgesia, results indicated that an activation in one area correlated with an activation in the other. Interestingly, they also assessed the individual fMRI connectivity, observing that the better connectivity a person has between these two regions, the better a placebo responder she will be. The most important implication, however, is the specific model of placebo responses that result from the hypothesis that these two diverse systems cooperate in eliciting placebo responses. In his view, placebo responses are best understood as the byproduct of aversive error-signalling processes that indicate a mismatch between what we expected the world to be and how the world eventually turned out to be. Thus, while for Benedetti and Wager, expectation and learning are two key components in explaining why we have placebo responses; to this view, Petrovic adds that it is only when such expectations fail to accurately predict external stimuli that such responses occur. Accordingly, placebo effects are just a consequence of the activity of an information processing system (the brain) trying to predict the world while minimising errors. On this model, whenever a placebo response occurs, the following elements are present: (i) some expectation about a determinate outcome (e.g., analgesia); (ii) an aversive stimuli (e.g., nociceptive signals that are still reaching the brain); (iii) a consequent error signal indicating the mismatch between (i) and (ii). Once (iii) occurs, there are two ways in which the system can minimise the error. One is to change the expectation for future occurrences; the other is to rely on top-down modulation (e.g., placebo analgesia by the activation of endogenous systems). The former is processed in the lateral OFC (expectation and error signals); the latter (top-down modulation) is processed in the ACC.

Understanding placebo responses as the byproduct of a brain seeking to minimise the mismatch between expectations (broadly conceived) and sensory signals has several theoretical and experimental implications. On the theoretical level, it means that it is wrong to maintain that there is a single placebo effect. Rather, placebo responses have several dimensions. In the case of pain, for example, we should expect that the system must be able to minimise errors not only by suppressing overestimated inputs (placebo analgesia) but also by increasing underestimated ones (nocebo hyperalgesia). Likewise, another relevant axis may be the one that goes from higher conscious expectations and cognitively mediated learning processes to more unconscious processes, whereby we learn to anticipate future situations, like in the case of classical conditioning. Finally, another relevant dimension will be the one that links internal stimuli (pain and emotions) with external stimuli (e.g., optical illusions). On the experimental side, instead, this model can be tested in several conditions to see whether or not the idea of placebo responses as ‘error-corrections’ holds true.

Petrovic and colleagues tested this hypothesis in schizophrenia and psychosis. The rationale for these experiments was the link between dopaminergic pathways, error signalling, and psychosis. In fact, the dopamine system is known to be extremely important not only in Parkinson’s disease—as stressed by Benedetti—and for reward processing—as noted by Wager—but and also in error signalling. Furthermore, dopamine is known to be very important in schizophrenia. But do psychosis and schizophrenia relate to placebo responses? Answering this question in controlled experiments is difficult because schizophrenic patients have much comorbidity and are on complex drug regimes. To overcome this difficulty, Petrovic and his colleagues assessed psychosis by using the Peter’s delusion index (PDI). This scale assumes that psychosis is a continuum condition, so that anyone can be defined as being more or less prone to psychosis. They tested in two experiments whether the aforementioned model of placebo effects could lead to predictions for these two conditions (research articles about these two studies are under review). The first experiment tackled expectation. They found a significant correlation; the higher the PDI score, the more the subject’s expectations were vivid. The second experiment tackled instead external sensory stimuli. Again, they found that the high PDI subjects perceive external signals more vividly. Together, these experiments provided preliminary evidence that high PDI subjects perceive more vividly their expectations about the world and/or external stimuli, hence indicating the possibility of an increased mismatch leading to the generation of averse-error signalling. This conclusion shows how knowledge in the field of placebo effects may provide a useful ground to generate models and hypotheses about other phenomena and conditions such as psychosis.

## Ted Kaptchuk: ‘Placebo effects in clinical practice’

The director of the PiPS, Ted Kaptchuk, delivered the fourth and final lecture of the event. In contrast with the previous talks centred on placebo mechanisms, Kaptchuk focused his lecture on what it means to study placebo effects in clinical practice. The talk was organised around three clinical trials to illustrate how the investigation of placebo responses entails at least two kinds of specificities. First, like any other clinical trial, studies aimed at studying placebo effects are not performed in a tightly controlled laboratory setting but on a target population of enrolled subjects. Second, they are not meant to study the specific effect of a drug but placebo responses. This latter aspect imposes unique constrains as to how the trial ought to be designed. Usually, when the aim of a trial is to study the specific effect of a drug, a placebo is used as a control. However, if the epistemic target of a trial becomes the study of placebo effects, what would be the control? How is the placebo effect to be studied in clinical trials?

To answer this question, it is first necessary to unpack the various components that are to be found in the control arm of a classic drug trial. For the purposes of the experiment, Kaptchuk divided the placebo components into two general kinds. The first type is represented by all the various effects that can be attributed to the performance of a therapeutic ritual, such as the act of ‘taking a pill’ or the general psychosocial context of clinical settings. The second kind is the doctor–patient relationship. In Kaptchuk’s view, the fact that this fundamental aspect of care delivery is generally listed among other confounding factors is an index of the pharmacocentric attitude driving medicine today. In addition to these two placebo components, other factors may explain the improvement observed in the control group; they are the natural course of the disease, the statistical phenomenon of regression to the mean, natural fluctuations, physical co-interventions, and purely psychological effects. To isolate placebo effects from this noise, several techniques can be deployed, such as the inclusion of a no-treatment group or a comparison between different placebos.

Against this background, a key issue in the field of placebo studies is whether it is possible to modulate placebo effects similar to a dose-dependence relationship. Is it possible to control placebo responses so that the magnitude of the effect varies in function of the quantity of the placebo medication prescribed? A study by Kaptchuk and colleagues investigated a closely related hypothesis, namely, whether there are diverse incremental components in placebo responses [17]. The experimental hypothesis was that it was possible to isolate two additive placebo components: (i) the one determined by the delivery of the therapy and (ii) the one determined by the interpersonal physician–patient relationship. The study was performed on 262 patients with irritable bowel syndrome (IBS). Participants were randomised into three groups. The first arm was the one of no treatment or waiting list and was meant to operate as the control for factors such as regression to the mean and spontaneous remission. In the second arm, patients received placebo acupuncture, but their relationship with the physicians was curtailed to a minimum—‘limited interaction’. Finally, in the third arm, patients received the same placebo acupuncture, but they found warm, confident, and emphatic physicians—‘augmented relationship’. Sham acupuncture in both groups was performed by the means of a ‘Steinberger needle’, a special device that looks and feels just like real acupuncture, except that it does not pierce the skin. Trial outcome was the same as the one required by the FDA to approve new drugs for IBS: global improvement scale, adequate relief of symptoms, symptom severity score, and quality of life. Results confirmed the initial hypothesis: for all four outcomes measured in the trial people in the limited interaction group reported more relief than the ones in the waitlist group, but less than the ones in the ‘augmented relationship group’. Improvements were significant: after three weeks, the felt improvement was 27% in the control group, 43% for the limited groups, and 62% in the augmented group. This latter had a magnitude of effects comparable with those of alosetron and tegaserod, the two most commonly prescribed drugs for this condition but which are known to have significant, even life-threatening, side effects. This study provided a first proof of principle that it is possible to isolate and combine diverse components of placebo effects and that non-specific effects can produce statistically and clinically significant outcomes.

One possible limitation to the conclusions of this study was that IBS symptoms and improvements are largely subjective. For this reason, the next trial presented by Kaptchuk investigated whether placebo responses could be experimentally induced in a condition such as asthma, for which the maximum forced expiratory volume (FEV) provides a reliable way of measuring a relatively simple objective outcome [18]. Are placebo interventions for asthma able to produce a quantifiable difference in FEV? To answer this question, the trial was designed as a double-blind, crossover study with 46 patients randomised to four arms: (i) active albuterol, (ii) placebo inhaler, (iii) sham acupuncture, and (iv) no-intervention group. To ensure that all volunteers had some asthma-related distress while being tested, they were taken off their asthma medication three days before the tests. The trial outcome was assessed in terms of FEV and subjective reports. Contrary to the initial hypothesis, people on albuterol reported a significant 20.1% FEV improvement, while people on placebo, sham acupuncture, and no intervention, all reported a virtually identical 7.5% improvement. This meant that placebo interventions were not able to modulate the objective outcome of the trial. Interestingly, however, a significant difference emerged in the subjective outcomes of the trial, with volunteers reporting a relief of 50% with the active drug, 45% with placebo, 46% with sham acupuncture, and 21% no intervention. Hence evidence from this study suggests that while placebo treatments cannot modify the FEV, nonetheless their clinical effects are relevant and comparable with those of albuterol.

Another lesson to be drawn from the asthma trial is that the underlying mechanisms of placebo responses are still poorly understood in conditions other than pain, depression, and Parkinson’s disease and a few others. In broadening our knowledge about placebo responses and their clinical implications, however, it is critical to address not only epistemological issues but also ethical worries. One of the key factors in explaining our ignorance of the placebo phenomenon is that many clinical trials cannot be conducted because they involve deception, making it difficult to obtain approval from IRB committees. This is especially true for oncology and other severe pathologies: while it may be acceptable to administer under controlled conditions a mild, local, and temporary painful stimulus on healthy volunteers, deceiving cancer patients for the purpose of testing a scientific hypothesis about a non-life-saving intervention does not sound morally justifiable. Ethics thus represents another important frontier in the future of the field of placebo studies; moral considerations—especially on the issue of deception—do make a real difference when it comes to deciding which trials can be conducted, and how they ought to be designed.

For this reason, a crucial question is whether deception is really necessary to elicit placebo responses. The theoretical rationale to question this assumption was provided by the findings of two qualitative studies conducted for the first IBS study. Based on their findings, two interesting facts emerged. First, many patients did not enrol because they expected to get better. Rather, they enrolled because they were desperate, mostly because they could not find an effective cure for their condition. This led to the question as to whether hope alone, rather than positive expectation elicited by manipulated expectations, could nonetheless determine a measurable placebo effect. Second, many participants in the first IBS study reported being suspicious as to their group allocation during the entire study. This could indicate that, even if people thought they were in the placebo arm, this belief still did not prevent the placebo response from occurring. Together, these two factors led to the question of whether open-label placebos are still effective despite the fact that the people taking them have been openly informed about their placebic nature. To test this hypothesis, in the third study presented by Kaptchuk, 80 volunteers with IBS were randomised to receive either no-treatment or a placebo pill twice a day [19]. People were recruited by telling them that a research group was performing an innovative study on mind–body interventions aimed at studying placebo effects. Subjects in the placebo arm were told that they had to ingest just placebo medications, and accordingly they received a bottle of cornstarch pills clearly labelled as ‘placebo’. Surprisingly, results indicated that taking two open-label placebos twice a day was significantly more effective than receiving no treatment for IBS. This study provided a first proof of principle that deception may not be necessary to elicit placebo responses, and that the performance of therapeutic ritual alone may be effective in bringing about health-relevant outcomes even when patients are fully informed that such a ritual is exclusively aimed at promoting placebo responses.

## Conclusion

Throughout the history of medicine, placebo effects have always acted as boundary makers. In the past, they marked the limits of what shamans, healers, and physicians could really do in the absence of known effective remedies. Then, in the age of science-based medicine, they indicated, in contrast, the boundaries of what were the legitimate targets of medical research: the underlying pathophysiological mechanisms of diseases and the specific effects of medical intervention that could be isolated and objectively measured in randomised double-blind placebo-controlled trials. Today, as the four lectures of the workshop ‘The science of placebo’ demonstrated, placebo effects may instead represent one of the most thriving frontiers of medical research and practice. Three major themes have emerged during this event, which let us speculate about how the proximate future of the field of placebo studies.

First, empirical results generated by the converging evidence of over 30 years of laboratory experiments, clinical trials, and neurocognitive sciences have now identified and partially explained several key mechanisms involved in placebo responses. Traditionally, the conditions in which placebo mechanisms have been investigated the most are pain, depression, and Parkinson’s disease, but an increasing number of clues indicate that psychosis and psychiatric disorders also may soon represent another major area for research on, or related to, placebo responses. This hints at an interesting shift in the field of placebo studies: while in the past, placebo effects were the target of empirical research, the more their mechanisms are understood and the more they are providing models to generate new empirical hypothesis about other conditions. Indirectly, this seems to suggest that placebo responses are not just a local phenomenon, but they may hint at something more profound and significant: a fundamental family of key mechanisms recruited in the experiences of ‘being sick’ and of ‘receiving a therapy’.

For these reasons, a second common theme among the four lectures was the necessity to better understand the possible interactions and interferences between conventional therapies and placebo effects or interventions. If expectations are crucial to elicit placebo responses, we should expect to encounter placebo-like effects in all clinical and research settings. No therapy and no experimental intervention are given in a complete cognitive and sensory vacuum, and thus there is always the possibility that what we observe at the end of a clinical trial or of a cycle of pharmacological interventions may be the combined product of specific and non-specific effects. Determining to what extent such effects add, interfere, or affect diverse outcomes in each condition is likely to be one of the core questions that will be tackled in the next decade by researchers.

Finally, the possibility of eventually translating the knowledge about placebo responses in better therapeutic regimes depends on how the evidence so far gathered will be corroborated by future experiments and trials. This, in turn, requires a major turnaround in how clinical research on placebo effects has been so far conceived, both from the epistemological and from the ethical point of view. From the introduction of new trial designs up to the elaboration of adequate controls, so far each major step in the science of placebo has been a lesson in the logic of scientific inquiry. Likely, the future of placebo studies will continue along this direction, with a new wave of empirical discoveries driven by a combination of innovations in trial design, more refined and integrated heuristic models, and ethically aware experimental protocols.

## Acknowledgements

This report was founded by a fellowship provided by the Fondazione Umberto Veronesi. I would like to thank Professor Fabrizio Benedetti and Professor Ted J Kaptchuk for the opportunity to participate in this event and Tommaso Bruni for his comments on an earlier draft of this report.
